# Establishment of stellate ganglion block in mice

**DOI:** 10.1186/s40001-024-01815-6

**Published:** 2024-04-04

**Authors:** Qirui Duan, Ying Zhou, Juan Zhi, Quanle Liu, Jin Xu, Dong Yang

**Affiliations:** Department of Anesthesia, Plastic Surgery Hospital, Chinese Academy of Medical Sciences and Peking Union Medical College, Shijingshan District, Beijing, 100144 China

**Keywords:** Stellate ganglion block, Mice, Horner’s syndrome, Autonomic nervous regulation (ANR), Animal model

## Abstract

**Background:**

There have been no reports on the successful implementation of stellate ganglion block (SGB) in mice.

**Objectives:**

This study aims to investigate a new method for implementing SGB in mice by placing them in a supine position with abducted upper limbs and touching the trachea and sternoclavicular joint with the hand.

**Methods:**

Fifty BABL/C mice, 8–10 weeks, were selected and randomly divided into four groups: control group (*n* = 5); SGB-R group (*n* = 15); SGB-L group (*n* = 15); and SGB-L + R (group *n* = 15). SGB was administered with 0.15% ropivacaine solution in a volume of 0.1 mL. The control group received equal volumes of saline. Horner's syndrome, heart rate, and complications such as brachial plexus block, vascular injury, pneumothorax, local anesthetic toxicity, and death were observed.

**Results:**

Horner's syndrome developed in 100% of SGB surviving mice; no difference was seen in the time to onset (100.4 ± 13.4 vs 96.7 ± 12.4, mean ± SD, seconds) and duration (264.1 ± 40.5 vs 296.3 ± 48.0, mean ± SD, min) of Horner's syndrome in the left and right SGB (***P*** > 0.05). Compared with the control group (722 [708–726], median [IQR], bpm), the heart rate was significantly slowed down in the right SGB (475 [451.5–491], median [IQR], bpm) (*P* < 0.05). While the heart rate was slowed down after performing the left SGB, the difference was not statistically significant (*P* > 0.05). The overall complication rate was 18.4%, with a brachial plexus block rate of 12.3%, a vascular injury rate of 4.6%, and a mortality rate of 1.5%, as well as no local anesthetic toxicity (includes bilateral implementation of SGB) or pneumothorax manifestations were found.

**Conclusions:**

This method allows for the successful implementation of SGB in a mouse model.

## Highlights

1. SGB successfully established in a mouse model. Also have a low complication rate.
2. Finding a new technical (placing them in a supine position with abducted upper limbs and touching the trachea and sternoclavicular joint with the hand. The needle is inserted next to the trachea, approximately 3–4 mm away and 2 mm above the sternoclavicular joint, in a 5° downward lateral declination) approach suitable for implementation in mice.

## Introduction

The Stellate Ganglion (SG) is a sympathetic ganglion that results from the fusion of the inferior cervical ganglion and the first thoracic ganglion. SG transmits sympathetic nerves to the upper limbs, head, neck, and heart [1]. Stellate Ganglion Block (SGB) is a clinical procedure that involves the injection of a local anesthetic (such as ropivacaine or bupivacaine) alongside the sympathetic ganglion. The technique has been in practice for over a century with a risk of serious complications estimated at 1.7 per 1,000 [2]. SGB is clinically useful not only in alleviating pain and vascular-related disorders within the innervated area [3], but also in managing hot flushes [4], post-traumatic disorders [5], cardiac arrhythmias [6], and immune-related conditions [7]. Notably, the clinical indications for stellate nerve blocks are broader in Japan than in Europe and the USA [7].

Due to the ubiquitous use of SGB in clinical applications, understanding its intrinsic mechanism of action is crucial. To this end, investigations in animal experiments are necessary. Establishing an effective and stable animal model that can serve as a basis for further research on SGB is crucial. Among the various animal models, rats are the most commonly used [10] and are similar to human SG in terms of anatomical structure and function [11]. Moreover, rats are often used in neurobiological studies [8, 10]. Conversely, mice possess immune characteristics similar to humans [9, 10] and are more suitable for tumor modeling [10].

Establishment of SGB in rat model was first reported in 2005 by Abdi S et al. [11] and later Gulcu N et al. [12] tibial modification was reported with 2009, both methods (blinded) using C7 as a bony marker in a rat model. No reports on the establishment of SGB in mice have been reported. It is worth noting that compared with rats, mice have a smaller body size and the anatomical structure of C7 is not obvious. Because of the smaller body size of mice and the less obvious anatomy of C7, the use of lateral puncture (Gulcu's method) or trans-posterior anterior puncture (Abdi's method) is not ideal because of the risk of scapular obstruction and the posterior access is positioned above the clavicle and distant from the SG. Because of the small size of the mice, the anatomical structures are adjacent to the smaller ones during ultrasound-guided real-time imaging, and the recognition rate is low, which reduces the advantages of ultrasound itself. Based on the above scholars' work, we propose the new technical hypothesis that SGB can be successfully established in vivo in a mouse model. It can serve as a reliable reference for SGB-related studies using mice as a research model. In this study, we propose a new blind method to establish SGB in a mouse model: according to the direction of the mouse neck fur is set as the midline, with the hand touching the trachea and the clavicle, at the angle of the two, the paratracheal paraspinal opening of about 3–4 mm, the clavicle is about 2 mm above the perpendicular to the horizontal position, the tip of the needle to the back of the lower part of the puncture, felt the first breakthrough, slightly backward, back to the bloodless injection of local anesthesia drug given. The purpose of this project was to investigate (i) whether the new SGB technique method can be successfully constructed in BABL/C mice; (ii) what effect exists on heart rate; (iii) whether the administration of 0.15% ropivacaine 0.1 ml can be used in bilateral SGB; and (iv) the complications and mortality of this technique in a mouse model.

## Methods

### Research approval

The study adhered to the guidelines established by the Research Animal Care Committee of the Plastic Surgery Hospital, Peking Union Medical College, Chinese Academy of Medical Sciences for animal care and experiments(Ethics approval number: 20220040614). The Institutional Animal Care and Use Committee of the Peking Union Medical College Plastic and Plastic Surgery Hospital granted approval for the experiments. The study also followed the ARRIVE guidelines for in vivo experiments in animal research and the laboratory animal practitioner certificate number was 1119121800169.

### Mice

The best way to estimate sample size in exploratory animal studies is the resource equation, which sets an acceptable range of degrees of freedom for error in an ANOVA. This applies to studies in which the outcome is a quantitative variable and is suitable for analysis of variance (ANOVA):

$$E = N - K = Kn - K\left( {n - 1} \right)$$

where 10 ≤ E ≤ 20, *N* is the total number, n is the sample size of each group, *K* is the number of treatment groups, and E is the difference between the two, the above formula can be converted as follows: *n* = E/*K* + 1. The minimum and maximum animal sample sizes required are: Min *N* = Min *n* × *K* = (10/*K* + 1) × *K*, Max *N* = Max *n* × *K* = (20/*K* + 1) × *K*. Because of the blind puncture method, and in the case of a blinded puncture, the minimum animal sample size is: Min *N* = Min n × *K* = (10/*K* + 1) × *K*, Max *N* = Max *n* × *K* = (20/*K* + 1) × *K*. This is suitable for research where results are a quantitative variable and where ANOVA is appropriate. It was a blinded puncture method, and also in the design of bilateral SGB, the optimal amount of drug was not further calculated, and in order to ensure statistical differences, the original sample size (*K* = 4, *N* = (20/*K* + 1) × *K* = 24) was enlarged by a factor of 1 in the present study [27].

A total of 50 BABL/C mice, aged 8–10 weeks and weighing between 20 and 24 g, were selected for this study. The animals were housed in a specified room wherein the constant temperature of 22 °C was maintained along with a 12-h light–dark schedule. Moreover, the mice had ad libitum access to food and water. The mice were allocated to one of the four experimental groups: control (*n* = 5); SGB-R (*n* = 15); SGB-L (*n* = 15); and SGB-L + R (*n* = 15).

### SGB localization

**Fig. 1:**
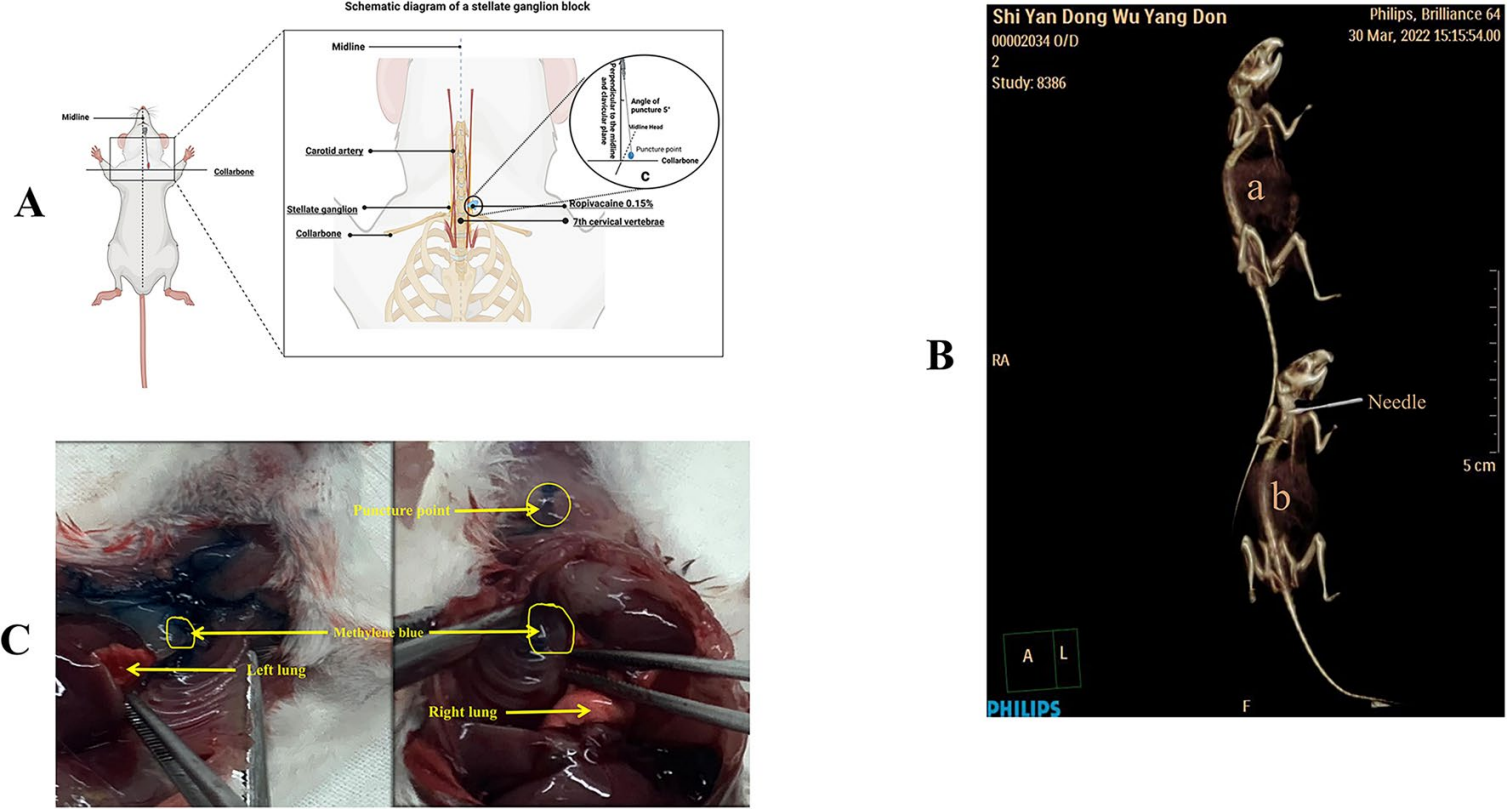
Mock-up of SGB performed in mice and the extent of diffusion observed by imaging localization and anatomical direct visualization. **A**: A mock-up of SGB in mice; **B**: The position of the needle tip when observing SGB under CT; **C**: An anatomical view of the extent of fluid diffusion following SGB after application of 0.1 ml of methylene blue as in A

Mice were anesthetized with sevoflurane (2.0 vol% for induction and 0.7–1.0 vol% for maintenance) without endotracheal intubation. After confirmation of complete sedation, mice were positioned in supine with upper limbs abducted to expose the neck and the anterior chest. Subsequently, the trachea and sternoclavicular joint were palpated at the point of confluence, where an indentation was made, which was located 3–4 mm parasternally away from the trachea and 2 mm above the sternoclavicular joint. The needle (Disposable intravenous infusion needle size 0.9 × 20 mm, Tianjin Hana Good Medical Material Co. Lot No.: 221014) was inserted at a downward lateral declination of 5°, followed by an injection of 0.1 ml of 0.15% ropivacaine (Ropivacaine Hydrochloride Injection, 10 ml: 75 mg, State Drug Administration H20113381, Jia Bo Pharmaceutical) without any aspiration of blood (refer to Fig. 1). Bilateral block was carried out using the same procedure on both sides, with the second block executed 30 min after the first one. The control group received a unilateral injection of 0.1 ml of saline. Two mice were selected at random to undergo CT scans to confirm the precision of the needle’s placement. After 24 h, 5% sevoflurane was administered to the mice, who were then placed in airtight bags at which point they were euthanized with carbon dioxide. Finally, all mice were administered 0.1 ml of methylene blue in the same manner, dissected, and observed for the spread of the drug after the injection to verify the accuracy of the location (refer to Fig. 1).

### Gold standard for success after SGB

Horner’s syndrome can be utilized as an indicator to assess the success of SGB, following Abdi S’s [11] method. The degree of ptosis is evaluated and recorded by a single blinded observer according to the following scale:—No ptosis: no difference in eye size between the two sides.  ± Unclear ptosis: uncertainty about the difference in eye size between the two sides. + Mild ptosis: barely noticeable upper eyelid drooping. +  + Moderate ptosis: eye size in the blocked side was more than half of that in the contralateral one. +  +  + Severe ptosis: eye size in the blocked side was less than half of that in the contralateral one. A return to + indicates the onset of SGB, corresponding to the observation of droopy eyelids, whereas a return to + signifies the loss of effect, which corresponds to the duration of droopy eyelids.

### Heart rate monitoring

Heart rate monitoring was performed by inducing sevoflurane again 15 min after administering SGB, and changes in heart rate were observed using the RM6240E from Chengdu Instrument Factory (www.scchengyi.com).

### Statistical analysis

R software (version 4.2.1) and its packages including ggplot2 [3.3.6], stats [4.2.1], and car [3.1–0] were used for statistical analysis. In the SGB-L + R group, two punctures per mouse were administered, and the total number of punctures was used as the denominator to compute complications. Appropriate statistical methods were selected based on the data format, such as the stats and car packages for statistical purposes, and the ggplot2 package for data visualization. Outlier analysis, normality test (Shapiro–Wilk normality test), variance Chi-square test (Levene’s test), as well as Kruskal–Wallis test were successively performed. Mean (Mean) ± standard deviation (SD) was used if the data were normally distributed, and if there were subgroups that did not satisfy normal distribution (*P* < 0.05), the choice was made to use a nonparametric test of the method and to express it in terms of the median [IQR]. We considered *P* < 0.05 as statistically significant.

## Results

### Horner's syndrome occurs in 100% of mice after SGB implementation

**Fig. 2:**
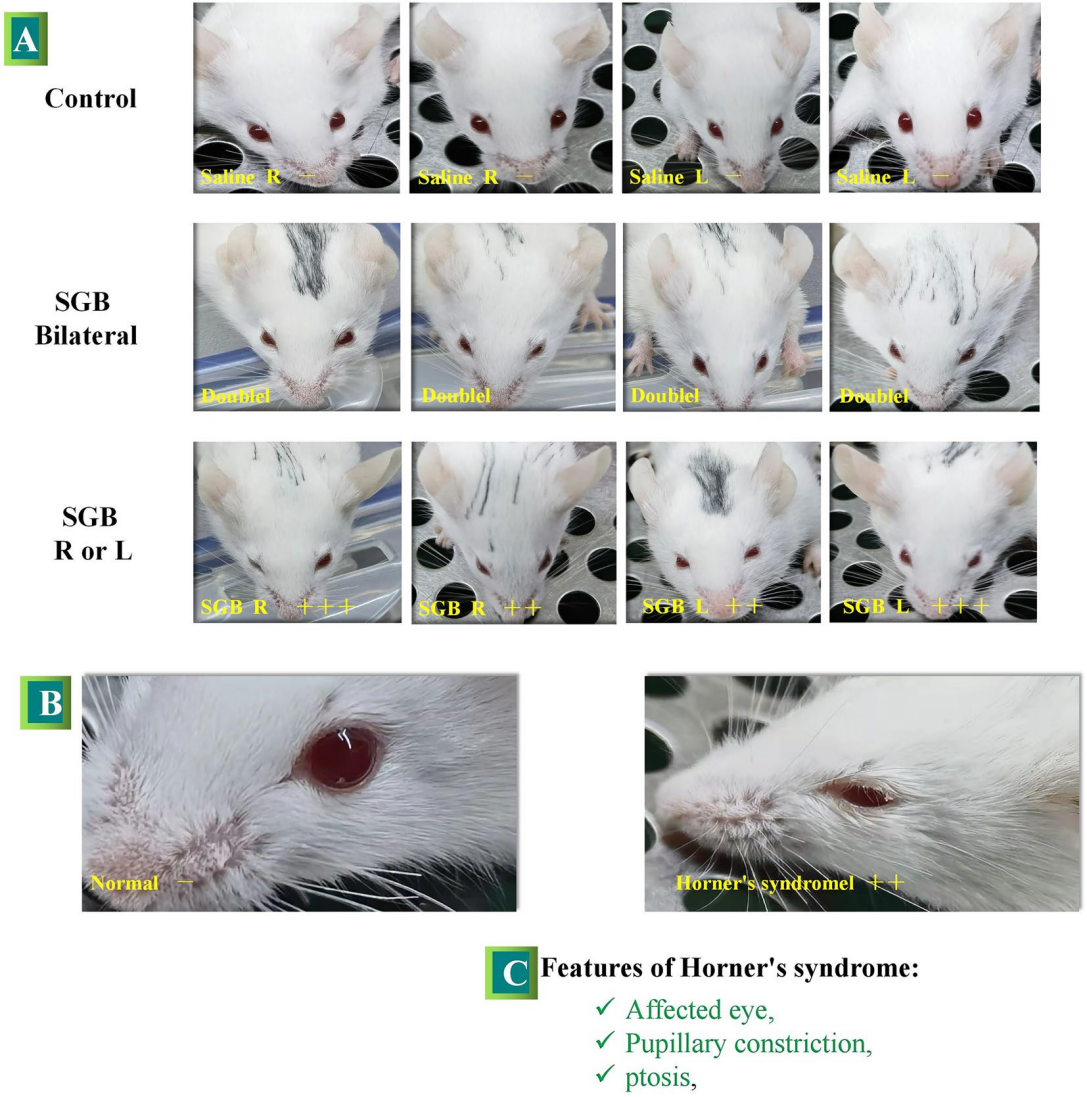
Horner’s syndrome after SGB in mice.** A:** overall view of Horner’s syndrome after control versus unilateral or bilateral SGB; **B:** comparison of Horner's syndrome +  + with normal eyelids; **C:** features of Horner’s syndrome that we can observe

**Fig. 3:**
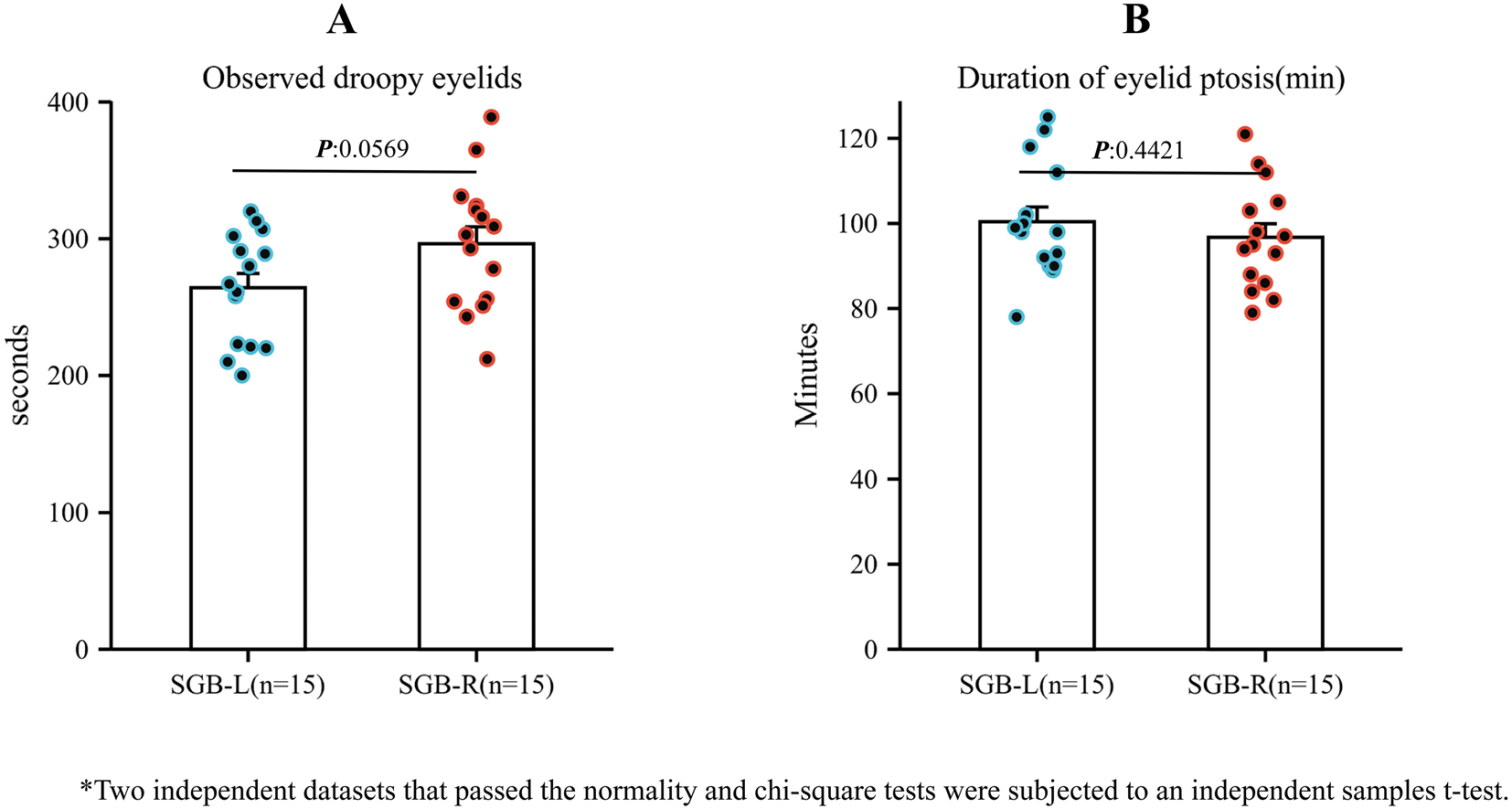
Time to onset of Horner's syndrome after left or right SGB and duration. **A:** time to onset of Horner's syndrome after left or right SGB (seconds); **B:** duration of Horner’s syndrome after left or right SGB (minutes)

Following sevoflurane cessation, surviving mice were awakened within 2 min. Horner's syndrome was observed in all mice around 4.5 min after awakening. The rate of Horner's syndrome after SGB was 100%, with observable eyelid ptosis (+ + and +  + +) rates of 74.1% and 25.9%, respectively. There were no significant differences in the observable eyelid ptosis or duration of eyelid ptosis between left-sided and right-sided SGB (Figs. 2 and 3; Table 1).

**Table 1:** Fghjjkk

	Total SGB	Control(n = 5)	SGB-L(n = 15)	SGB-R(n = 15)	SGB-L + R(n = 15 × 2)
Observation indicators					
Horner’s syndrome	58	0	15	15	14 × 2
− (n, %)	0	0	0	0	0
± (n, %)	0	0	0	0	0
+ (n, %)	0	0	0	0	0
+ + (n, %)	43 (74.1%)	0	10 (66.7%)	11 (73.3%)	22 (78.6%)
+ + + (n, %)	15 (25.9%)	0	5 (33.3%)	4 (26.7%)	6 (21.4%)
Observed droopy eyelids (mean ± SD, seconds)	–	–	264.1 ± 40.5	296.3 ± 48.0^a^	–
Duration of droopy eyelids (mean ± SD, min)	–	–	100.4 ± 13.4	96.7 ± 12.4^b^	–
Heart rate (Median [IQR], bpm)	–	722 [708–726]	507 [491–527.5]	475 [451.5–491]	410.5 [381.5–429.25]
Complications					
Total	12/(35 + 15 × 2) (18.4%)	1 (20%)	2 (13.3%)	2 (13.3%)	7 (23.3%)
Brachial plexus nerve block (n, %)	8/(35 + 15 × 2)(12.3%)	0	2 (13.3%)	1 (6.7%)	5 (16.7%)
Vascular injury (n,%)	3/(35 + 15 × 2)(4.6%)	1 (20%)	0	1 (6.7%)	1 (3.3%)
Pneumothorax (n,%)	0	0	0	0	0
Local anesthetic intoxication (n,%)	0	0	0	0	0
Death (n, %)	1/(35 + 15 × 2)(1.5%)	0	0	0	1 (3.3%)

Basic information on the establishment of SGB in mouse models

^a^SGB-R group compared to SGB-L group, *T* test, *p* = 0.0569; b: SGB-R group compared to SGB-L group, *T* test, *p* = 0.4421;

### Heart rate reduction after SGB implementation in mouse models

**Fig. 4:**
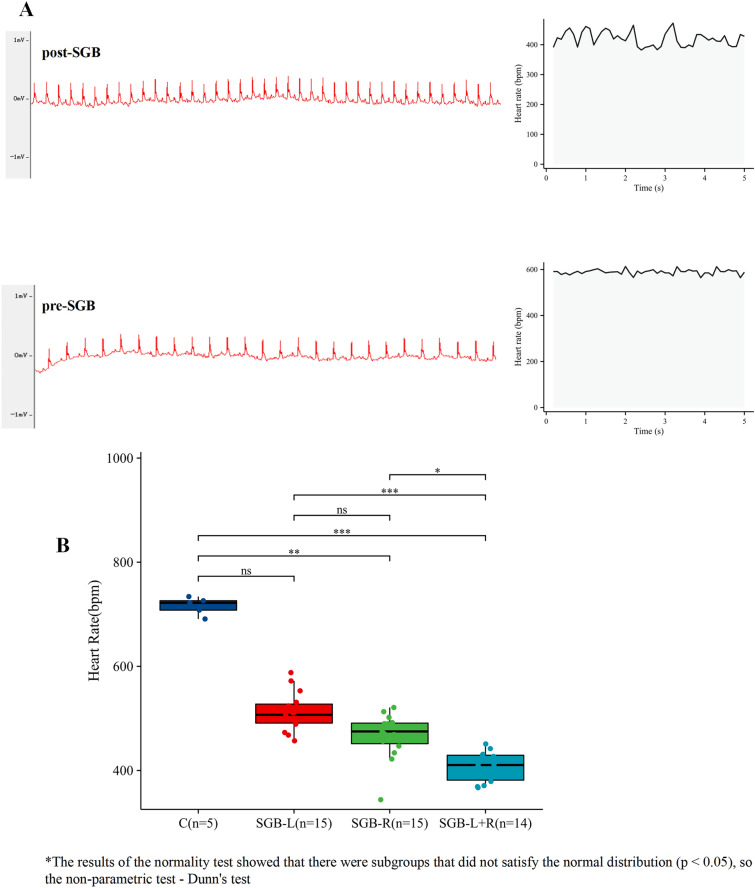
ECG and heart rate changes in mice after SGB. **A:** ECG and 5 s heart rate changes in the same mice after SGB vs. control. **B:** comparison of heart rates in each group after SGB; SGB-L vs. control, ***P*** = 0.4330; SGB-R vs. Control, ***P*** = 0.0100; SGB-L + R vs. Control, ***P*** = 2.09e−06; SGB-L vs SGB-R, ***P*** = 0.3415; SGB-L vs SGB-L + R, ***P*** = 2.04e−05; SGB-R vs SGB-L + R, ***P*** = 0.0332.*P < 0.05, **P < 0.01, ***P < 0.001

All surviving mice were subjected to heart rate monitoring 15 min after the implementation of the SGB intervention. Compared with the control group, the heart rates of both the SGB-R group and the SGB-L + R mice were significantly lower (*p* < 0.01); the heart rates of the mice in the SGB-L group were lower, and the difference was not statistically significant (*p* > 0.05). Compared with the SGB-R or SGB-L groups, the heart rate of SGB-L + R mice in the SGB-L + R group, in which bilateral SGB intervention was implemented, was further reduced, and the difference was statistically significant (*p* < 0.05).The difference in the heart rate of the SGB-R group was not statistically significant when compared with that of the SGB-L group (*p* > 0.05) (Fig. 4; Table 1).

### Incidence of brachial plexus block following SGB in mice is approximately 12%

Following SGB, mice were classified as having a brachial plexus block if they exhibited difficulty moving the upper limb on the side of the block or were unable to use that side of the limb for walking and resumed normal activity after 24 h. Among all surviving mice, eight cases of brachial plexus block were observed, with an overall incidence of 12.3%. Two cases occurred in the SGB-L group (13.3%), one in the SGB-R group (6.7%), and five in the SGB-L + R group (16.7%) (as shown in Table 1).

### No cases of local anesthetic intoxication observed following SGB in mouse models

Local anesthetic intoxication reactions were judged based on the presence of symptoms, such as convulsions, dyspnea, limb stiffness, nausea, or vomiting after SGB. None of the surviving mice exhibited such symptoms. In addition, there were no cases of local anesthetic intoxication following bilateral SGB (as shown in Table 1).

### Vascular damage rate in mice following SGB is less than 5%

Vascular damage was defined as any bright red blood seen emerging or oozing after the needle tip was removed. After SGB implementation, three cases of vascular damage were observed, resulting in a rate of 4.6%. There was one case each in the SGB-L group, SGB-R group 1, and SGB-L + R group (as shown in Table 1).

### Mortality rate of SGB in mice is less than 2%

A total of 65 punctures were performed during the study. In one instance, in the SGB-L + R group, the needle puncture was approximately 3 mm from the midline during the second attempt. Bright red blood was observed after the needle was removed without any blood administration. Death occurred about 30 s later. Therefore, the total mortality rate was calculated to be 1.5% (1/[35 + 15 × 2]).

## Discussion

In comparison with rats, mice are cost-effective, exhibit little individual variation, and are commonly used in immune-related animal studies and tumor models [8–10]. Although SGB technology has been applied to rats [11, 12], this is the first description of SGB technology in mice. Like in rats [14], the stellate ganglion of mice is composed of the inferior cervical ganglion and the first-to-third thoracic ganglia [13, 14], located between C7 and T1 [13, 15]. In mice, the stellate ganglion is situated behind the carotid bifurcation within the region of the first rib and longissimus cervicis [14]. Although the anatomical location of the stellate ganglia is similar in mice and rats, the number and content of cells, certain transmitters, and eyelid and heart regulation are different in both [14–16]. Nevertheless, the presence of ptosis in both mice and rats indicates sympathetic blockade [11–15]. Since the Salahadin Abdi [11] and Nebahat Gulcu [12] techniques for SGB implementation in rats are not feasible in mice due to their smaller size and body proportion, we adopted a new method by supine anaesthetization with fully extended neck and anterior thorax, thereby indicating successful SGB via the gold standard Horner syndrome. However, differences in the innervation of eyelids by the stellate ganglion between rats and mice [16] may also influence the variable outcomes of Horner's syndrome in both rodents due to pharmacodynamic effects. As the sympathetic ganglion, the SG reduces sympathetic activity, thus resulting in a slower heart rate; however, some reports suggest that the cervical ganglion and vagus nerve travel within the same vascular sheath [17]. Consequently, although the vagus nerve may also undergo blockage during SGB administration, no detrusor effect was observed, and the degree of blockade may be greater for the sympathetic than the vagus nerve, possibly causing a greater sympathetic effect than the vagal effect [22]. Of course, there is also a mix of sympathetic and vagal fibers, the stellate ganglion also contains vagus nerve fibers [22].etc. It should be clear to us that crosstalk between the sympathetic and vagus nerves is very complex. In the current study, there was a decrease in heart rate with a single implementation of left-sided SGB, but there was no statistical difference. In contrast, the effect on heart rate was more significant (*P* < 0.05) when the right SGB was performed in a single session. Currently, there is no relevant description of the effect of SGB on heart rate in a mouse model. A study based on the analysis of heart rate variability power spectra through SGB administration in 20 young healthy volunteers found that right SGB not only reduced the lnLF (low-frequency power, mainly on sympathetic nerves) component from 6.55 ± 0.84 to 5.77 ± 0.47, but also the lnHF (high-frequency power, mainly on parasympathetic nerves) component from 4.40 ± 0.95 decreased to 3.42 ± 1.12 (*p* < 0.05), while no significant change was observed on the left side. The following conclusions were drawn: (1) autonomic innervation to the sinus node is predominantly via the right SG; (2) pharmacologic right SGB attenuates not only sympathetic but also parasympathetic activity; and (3) the reduced sympathetic and parasympathetic influences on the sinus node may inconsistently counterbalance and alter the RR interval after right SGB [18]. Other studies have also shown different centers of gravity for left (providing ventricular coronary arteries and myocardium) and right (responsible for heart rate control of the sinus node) SGB fiber regulation [22]. The above conclusions, although derived from human experiments, may also explain the lack of difference in heart rate between the SGB-L group and control in this study. In awake intubated patients, heart rate variability due to intubation response may be reduced after implementation of right-sided SGB [19]. However, in patients with supraventricular tachycardia, the human conduction system and the Kent bundle were found to be dominated by SG from the left side [20]. Furthermore, in terms of regulating the autonomic nervous system, there is a large difference in the modulation of sympathetic and parasympathetic nerves by the left and right SGB [21]. Notably, the effect on hemodynamics is small regardless of whether left- or right-sided SGB is implemented [22]^.^

The implementation of bilateral SGB has also been reported in clinical practice [23–25], but it is not common. In contrast, the present study was designed with a bilateral SGB block for three purposes: first, to observe whether the dose of local anesthetics in the present study showed toxic responses under doubling; second, to provide a basis for future mouse models that may require bilateral block; and third, to compensate to some extent for the relative inadequacy of the design of the study (to study the appropriate dose of the drug and the dosage to achieve a precise SGB).

In regard to complications, we calculated the total number of SGBs and recorded brachial plexus blocks or injuries, vascular injuries, pneumothorax, local anesthetic poisoning, and deaths. Notably, our approach had a similar rate of brachial plexus block to Lin's study in rats [26]; our method had a significantly lower rate of vascular injury, pneumothorax, and mortality than Lin's blinded puncture experiment [26]. Notably, brachial plexus blocks are acceptable temporary side effects. No symptoms associated with local anesthetic toxicity were identified, although one death occurred immediately after puncture, which was not attributed to local anesthetic toxicity, as it occurred 30 s after the block. Limitations of this study include the non-correlation of the dose and volume of the drug administered with pathophysiological and pharmacological variables. Moreover, we did not undertake additional analysis of heart rhythm and temperature changes despite monitoring heart rates. Another drawback was the behavioral observation of complications such as the brachial plexus block and local anesthetic poisoning without corresponding analysis of pathophysiological and pharmacological aspects. The causes of death were not further analyzed. For future studies, the precise dose and volume of the drugs and monitoring changes in heart rhythm and body temperature should be taken into account to ensure the presence and integrity of sympathetic blockade. While this study presents a new approach for SGB implementation in mice, it does not address other effects of modulation by the stellate ganglion. Hopefully, this study will offer technical assistance and support for future SGB studies in mice.

## Limitations

This study reports on the implementation method of SGB in mice and the gold standard for successful SGB, as well as associated complications. However, the experimental design did not correlate the dose and volume of the drug, omitting a crucial aspect of the experiment. In addition, although heart rate was monitored, no further analysis of changes in heart rhythm and body temperature were carried out. The complications of brachial plexus block and local anesthetic poisoning reactions were solely observed behaviorally, without correlating the pathophysiological and pharmacological perspectives, making this approach inadequate. Furthermore, the specific reasons for the deaths occurring after puncture were not analyzed. Future studies must focus on precise anesthesia through investigating appropriate doses and volumes of drugs. The monitoring should include analysis of changes in heart rhythm and temperature to ensure the presence and integrity of sympathetic blockade. This study provides a new approach to the modulation of the organism by the stellate ganglion; however, it does not discuss other potential aspects. The study results can provide technical support and guidance to future studies in SGB in mice.

## Conclusion

The present study utilized a new method of SGB, which involves positioning the mice supinely with the upper limbs abducted, the neck and anterior thorax fully exposed, and hand contact with the trachea and sternoclavicular joint. Additionally, the paratracheal opening was approximately 3–4 mm at an angle of 2 mm above the sternoclavicular joint, with the needle tip pierced downwards at a 5° lateral deviation. This method was successful in achieving Horner's syndrome and heart rate reduction in mice, with low complications and mortality.

## Data Availability

All data generated or analysed during this study are included in this published article [and its supplementary information files].

## Abbreviations

SGB: Stellate ganglion block
C7: 7Th cervical vertebra
L: Left SGB
R: Right SGB
